# Genetic liability of gut microbiota for idiopathic pulmonary fibrosis and lung function: a two-sample Mendelian randomization study

**DOI:** 10.3389/fcimb.2024.1348685

**Published:** 2024-05-22

**Authors:** Yuan Ren, Yao Zhang, Yanan Cheng, Hao Qin, Hui Zhao

**Affiliations:** ^1^ Department of Pulmonary and Critical Care Medicine, The Second Hospital of Shanxi Medical University, Taiyuan, China; ^2^ The Second Clinical Mediccal college, Shanxi Medical University, Taiyuan, China

**Keywords:** Mendelian randomization, idiopathic pulmonary fibrosis, gut microbiota, lung function, fatty acids

## Abstract

**Background:**

The microbiota-gut-lung axis has elucidated a potential association between gut microbiota and idiopathic pulmonary fibrosis (IPF). However, there is a paucity of population-level studies with providing robust evidence for establishing causality. This two-sample Mendelian randomization (MR) analysis aimed to investigate the causal relationship between the gut microbiota and IPF as well as lung function.

**Materials and methods:**

Adhering to Mendel’s principle of inheritance, this MR analysis utilized summary-level data from respective genome-wide association studies (GWAS) involving 211 gut microbial taxa, IPF, and lung function indicators such as FEV_1_, FVC, and FEV_1_/FVC. A bidirectional two-sample MR design was employed, utilizing multiple MR analysis methods, including inverse variance-weighted (IVW), weighted median, MR-Egger, and weighted mode. Multivariable MR (MVMR) was used to uncover mediating factors connecting the exposure and outcome. Additionally, comprehensive sensitivity analyses were conducted to ensure the robustness of the results.

**Results:**

The MR results confirmed four taxa were found causally associated with the risk of IPF. *Order Bifidobacteriales* (OR=0.773, 95% CI: 0.610–0.979, p=0.033), *Family Bifidobacteriaceae* (OR=0.773, 95% CI: 0.610–0.979, p=0.033), and *Genus RuminococcaceaeUCG009* (OR=0.793, 95% CI: 0.652–0.965, p=0.020) exerted protective effects on IPF, while *Genus Coprococcus2* (OR=1.349, 95% CI: 1.021–1.783, p=0.035) promote the development of IPF. Several taxa were causally associated with lung function, with those in *Class Deltaproteobacteria, Order Desulfovibrionales, Family Desulfovibrionaceae, Class Verrucomicrobiae*, *Order Verrucomicrobiales* and *Family Verrucomicrobiaceae* being the most prominent beneficial microbiota, while those in *Family Lachnospiraceae, Genus Oscillospira*, and *Genus Parasutterella* were associated with impaired lung function. As for the reverse analysis, MR results confirmed the effects of FEV_1_ and FVC on the increased abundance of six taxa (*Phylum Actinobacteria, Class Actinobacteria, Order Bifidobacteriales, Family Bifidobacteriaceae, Genus Bifidobacterium*, and *Genus Ruminiclostridium9*) with a boosted level of evidence. MVMR suggested monounsaturated fatty acids, total fatty acids, saturated fatty acids, and ratio of omega-6 fatty acids to total fatty acids as potential mediating factors in the genetic association between gut microbiota and IPF.

**Conclusion:**

The current study suggested the casual effects of the specific gut microbes on the risk of IPF and lung function. In turn, lung function also exerted a positive role in some gut microbes. A reasonable dietary intake of lipid substances has a certain protective effect against the occurrence and progression of IPF. This study provides novel insights into the potential role of gut microbiota in IPF and indicates a possible gut microbiota-mediated mechanism for the prevention of IPF.

## 1 Introduction

The gut microbiota refers to the collection of various microbial communities that parasitize the host’s gastrointestinal tract, comprised of bacteria, fungi, viruses, archaea, and protozoa (Lynch and Pedersen, 2016). Typically, these microorganisms exist in a symbiotic relationship with the human host and play a crucial role in maintaining immune function and balance through interactions with the host’s immune system (La Barbera et al., 2022). The Lung-Gut axis is the concept of the mutual connection and interaction between the lungs and the gut, as these two organs are the largest surface organs with highly vascularized and immunologically active tissues. With their exposure to various external environmental challenges, including pathogenic microorganisms, harmful gases, and particulate matter, there is growing attention on the importance of the Lung-Gut Axis in impacting disease states in various ways, such as immune system communication (McAleer and Kolls, 2018), lung inflammation, and gut mucosal barrier. Changes in the gut microbiota have been linked to respiratory conditions like asthma (Barcik et al., 2020), chronic obstructive pulmonary disease (Bowerman et al., 2020), connective tissue-associated interstitial lung diseases (Salisbury et al., 2017), and acute lung injury (Kapur et al., 2018). Therefore, some gut microbial communities can serve as biomarkers for lung diseases.

Idiopathic pulmonary fibrosis (IPF) is a rare and severe chronic respiratory disease characterized by progressive interstitial lung damage and declining lung function. Symptoms of IPF include fatigue, shortness of breath during physical activity, and a persistent dry cough. This condition ultimately leads to organ failure and death. The exact cause of IPF is not fully understood, but various risk factors are thought to contribute to its development, including intrinsic factors such as genetics, aging, gender, and lung microbiota, as well as extrinsic factors such as smoking, environmental exposures, and air pollution. The incidence of IPF has been steadily increasing, likely due to factors such as aging populations and deteriorating air quality (Harari et al., 2020). IPF has an insidious onset and is often diagnosed at an advanced stage, resulting in a median survival of only 3.8 years (Neumark et al., 2020). Unfortunately, there is currently a lack of reliable diagnostic approaches in the early phase of the disease and effective treatments. Recent research suggests that dysbiosis of the gut microbiota might be associated with the progression of IPF (Ntolios et al., 2021; Quan et al., 2022). During acute exacerbation of the disease, patients often exhibit a higher microbial burden in their lungs. While there is a known correlation between the lung microbiome and disease severity, as well as the risk of disease progression and mortality, a causal relationship has yet to be established (Ntolios et al., 2021).

The link between the microbial community and IPF can be connected through related metabolites, such as fatty acids. Regarding bacterial metabolites, fatty acids may closely associate with pathophysiological processes such as mitochondrial dysfunction, functional impairment, oxidative stress, and affecting the progression of IPF (Wu et al., 2022).

Mendelian randomization (MR) analysis is a powerful tool that can leverage pre-existing aggregated data from genome-wide association studies (GWAS) to investigate the associations between complex traits and millions of molecular marker single nucleotide polymorphisms (SNPs). By using genetic variants as instrumental variables (IVs), MR can infer causality between exposure and its effect while mitigating the influence of unobserved confounding factors. Moreover, the accurate measurement of genetic variation in MR is not susceptible to measurement errors. Importantly, genetic variants are randomly allocated before birth (Birney, 2022), which aligns with the chronological order of causal timing and minimizes issues of reverse causality. As a result, MR has become an increasingly important technique in epidemiological research focusing on causal inference (de Leeuw et al., 2022). Through comparative analyses of genetic variants, MR can identify those that influence complex traits (Swerdlow et al., 2016). It offers an advantage over traditional observational studies, as it provides a robust framework for inferring causality while minimizing the risk of bias from confounding variables.

## 2 Materials and methods

### 2.1 Study design

**Figure 1:**
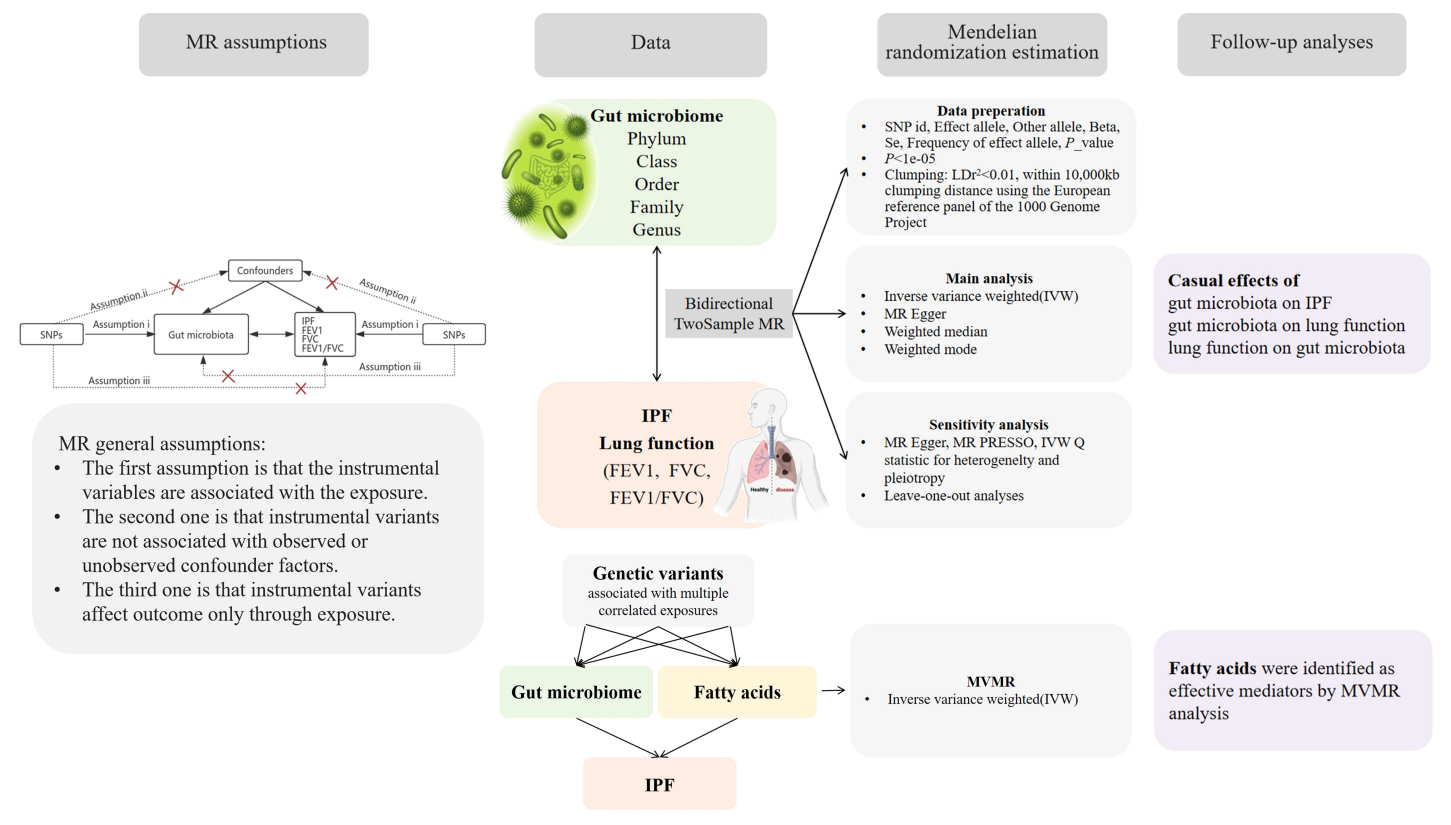
A bidirectional two-sample MR model was used to evaluate the causal relationships between exposure and outcome.

**Figure 1** provides an overview of the study design and assumptions underlying MR research. In MR studies, IVs must satisfy three key assumptions (Emdin et al., 2017). Assumption 1 requires that the chosen genetic variants, proposed as instrumental variables, are reliably associated with the risk factor under investigation. Assumption 2 states that the selected genetic variants should not possess any associations with potential confounding factors. Assumption 3 states that the genetic variants selected as IVs should influence the outcome risk solely through the risk factor of interest, rather than through alternative pathways.

### 2.2 Data sources of gut microbiome and fatty acids

The gut microbiota data used in this study were sourced from the MiBioGen consortium, which includes genome-wide genotypes and 16S fecal microbiome data from 18,340 participants across 24 cohorts, featuring 5,717,754 SNPs for a total of 211 taxa that encompass 9 phyla, 16 classes, 20 orders, 35 families, and 131 genera (Kurilshikov et al., 2021). Furthermore, we also sought to explore the potential role of fatty acids in the biological pathway of the gut microbiota to IPF. Several important fatty acids indicators were identified (including monounsaturated fatty acids, omega-3 fatty acids, omega-6 fatty acids, polyunsaturated fatty acids, ratio of docosahexaenoic acid to total fatty acids, ratio of linoleic acid to total fatty acids, ratio of monounsaturated fatty acids to total fatty acids, ratio of omega-3 fatty acids to total fatty acids, ratio of omega-6 fatty acids to omega-3 fatty acids, ratio of omega-6 fatty acids to total fatty acids, ratio of polyunsaturated fatty acids to monounsaturated fatty acids, ratio of polyunsaturated fatty acids to total fatty acids, ratio of saturated fatty acids to total fatty acids, saturated fatty acids, and total fatty acids). The GWAS data for fatty acids were extracted from MRC-IEU OpenGWAS project (https://gwas.mrcieu.ac.uk/). Detailed information is shown in **Table 1**. To ensure minimal overlap with IPF studies, we specifically selected studies with no or minimal sample overlap. After calculation, we obtained a maximum overlapping rate of <10%, which may not have been sufficient to affect our results (Rees et al., 2017).

**Table 1:** Characteristics of the GWASs used for Analyses.

Trait		Data resource	PubMed Identification	Population	Sample size
Gut microbiome	Phylum	MiBioGen consortium (www.mibiogen.org)	33462485	European (16 cohorts, N=13,266), Middle-Eastern (1 cohort, N=481), East Asian (1 cohort, N=811), American Hispanic/Latin (1 cohort, N=1097), African American (1 cohort, N=114) multi-ancestry (4 cohorts, N=2571)	18,340 participants
	Class				
	Order				
	Family				
	Genus				
IPF	Based on the guidelines established by the American Thoracic Society and the European Respiratory Society	The Collaborative Group of genetic studies of IPF (https://github.com/genomicsITER/PFgenetics#study2)	35688625	European	4,125 cases and 20,464 controls
Lung function	FEV1	GWAS Catalog (https://www.ebi.ac.uk/gwas/) under the accession codes GCST90292609, GCST90292610, and GCST90292611	36914875	European	475,645 participants
	FVC				
	FEV1/FVC				
Fatty acids	Fifteen fatty acids indicators	https://gwas.mrcieu.ac.uk/	NA	European	114,999 participants

NA, not applicable.

### 2.3 Data sources of IPF and lung function

The data relevant to IPF were obtained from the GWAS study led by Richard J. Allen (Allen et al., 2022), which included a cohort of 4,125 cases and 20,464 controls of European ancestry from diverse regions, such as the USA, UK, and Spain. The diagnosis of IPF was conducted based on the guidelines established by the American Thoracic Society and the European Respiratory Society (Raghu et al., 2011, Raghu et al., 2018). Furthermore, the largest multi-ancestry GWAS of lung function to date, involving 475,645 participants in Europe, is accessible on the GWAS Catalog (https://www.ebi.ac.uk/gwas/) under the accession codes GCST90292609, GCST90292610, and GCST90292611 (Shrine et al., 2023).

### 2.4 Instrument selection and data harmonization

Given the limited number of available SNPs, we selected SNPs significantly related to the gut microbiota with a loose cutoff of p < 1e^-5^. Then, significant SNPs were clumped within 10,000 kb at the level of linkage disequilibrium (LD) r^2^ = 0.01 using the European reference panel of the 1000 Genome Project. In reverse MR analyses, independent SNPs were selected by LD (r^2^ < 0.01 within 250-kb clumping distance, based on the European reference panel of the 1000 Genome Project) at a compromised significant level (1e^-5^) due to the relatively insufficient variables.

### 2.5 Statistical analysis

A two-sample MR approach was implemented to investigate the causal associations between 211 microbial taxa and IPF as well as lung function. To align the effects, all SNPs were harmonized between the exposure (microbial taxa) and the outcome (IPF and lung function) based on alleles. SNPs associated with reverse causality were removed using the Steiger_filtering test (Hemani et al., 2017).

The inverse-variance weighted (IVW) method was chosen as the primary approach to estimate the total causal effect of the exposure on the outcome (Slob and Burgess, 2020). In this method, two or more IVs were combined by calculating the weighted average variance, with each IV’s weight determined as the reciprocal of the variance of the effect estimate. To complement the IVW method, we performed additional analyses including MR Egger, weighted median, and weighted mode. MR Egger allowed us to investigate the mean horizontal pleiotropic effect across instrumental variables (Burgess and Thompson, 2017). The weighted median approach generated robust estimates of the causal effect in situations where at least half the weight was derived from valid instruments, minimizing the impact of instrumental outliers (Bowden et al., 2016). Similarly, the weighted mode method assumed that the frequently observed association estimate was not influenced by pleiotropy and thus accurately reflected the true causal effect (Hartwig et al., 2017). Causal effect estimates were reported as β and Odds Ratios (OR) (OR = expβ).

We estimated the proportion of trait variance explained by the genetic instruments identified by the formula R2 = (2β^2^×EAF×(1-EAF))/(2β^2^×EAF×(1-EAF)+2N×EAF×(1-EAF)×SE^2^), where EAF represents the effect allele frequency, β denotes the effect size of SNP in the exposure GWAS, SE refers to the standard error, and N represents the sample size of the exposure GWAS. Instrument strength was assessed using the F statistic, where F = (R^2^ × (N-2))/(1-R^2^) (Palmer et al., 2012; Morales Berstein et al., 2022). SNPs having an F value < 10 were excluded due to their weak statistical strength. These analyses were conducted to provide a comprehensive evaluation of the causal relationships between microbial taxa and IPF, thereby enhancing the robustness and reliability of our study findings.

In an effort to identify potential vertical pleiotropic pathways that may arise from specific microbiotic metabolites, multivariable Mendelian randomization (MVMR) analyses were performed using MVMR_IVW to estimate the causal effect of specific gut microbiota on IPF after adjusting for fatty acids (Burgess and Thompson, 2015). In addition, the mr_pleiotropy_test (Verbanck et al., 2018) and IVW Q statistic (Greco et al., 2015) were utilized to identify horizontal pleiotropic outliers and quantify heterogeneity. The absence of pleiotropic effects was determined if the intercept did not significantly deviate from 0 (p>0.05). Furthermore, a leave-one-out analysis was conducted to identify potentially influential SNPs. The usage and interpretation of our MR study adhere to the STROBE-MR (Strengthening the Reporting of Observational Studies in Epidemiology-Mendelian Randomization) checklist (Skrivankova et al., 2021) (**Supplementary Table S1**).

All statistical analyses were undertaken using the “TwoSampleMR”, “MR-PRESSO”, and “MVMR” packages in R version 4.3.1 (http://www.r-project.org/), and a two-tailed p-value of less than 0.05 was considered statistically significant.

## 3 Results

### 3.1 Overview

After screening for SNPs linked with exposure and removing LD, 2,875 SNPs from 211 taxa were employed as IVs. After harmonizing exposure and outcome alleles, all SNPs from various taxa performing MR analysis were shown in **Supplementary Table S2**. The conclusive findings between gut microbiota and IPF and lung function were summarized in **Tables 2**–**6**.

**Table 2:** Associations of genetic predisposition to gut microbiome with the risk of IPF.

Exposure	Outcome	n(SNP)	Method	OR(95%CI)	*P*	*P* for IVW Q statistic	*P* for MR-Egger intercept test
Order Bifidobacteriales	IPF	16	MR Egger	0.745(0.253,2.195)	0.602	0.672	0.948
			Weighted median	0.933(0.678,1.284)	0.670		
			Inverse variance weighted	0.773(0.610,0.979)	0.033		
			Weighted mode	0.968(0.585,1.602)	0.901		
Family Bifidobacteriaceae	IPF	16	MR Egger	0.745(0.253,2.195)	0.602	0.637	0.948
			Weighted median	0.932(0.674,1.291)	0.675		
			Inverse variance weighted	0.773(0.610,0.979)	0.033		
			Weighted mode	0.968(0.593,1.580)	0.898		
Genus Coprococcus2	IPF	12	MR Egger	1.090(0.248,4.792)	0.911	0.830	0.779
			Weighted median	1.238(0.859,1.785)	0.253		
			Inverse variance weighted	1.349(1.021,1.783)	0.035		
			Weighted mode	1.219(0.695,2.137)	0.504		
Genus RuminococcaceaeUCG009	IPF	13	MR Egger	0.898(0.365,2.214)	0.820	0.381	0.787
			Weighted median	0.791(0.604,1.035)	0.088		
			Inverse variance weighted	0.793(0.652,0.965)	0.020		
			Weighted mode	0.988(0.626,1.557)	0.958		

**Table 3:** Associations of genetic predisposition to gut microbiome with the value of FEV1.

Exposure	Outcome	n(SNP)	Method	β	*SE*	*P*	*P* for IVW Q statistic	*P* for MR-Egger intercept test
Class Deltaproteobacteria	FEV1	14	MR Egger	0.012	0.039	0.767	0.035	0.695
			Weighted median	0.021	0.015	0.155		
			Inverse variance weighted	0.027	0.013	0.042		
			Weighted mode	0.014	0.019	0.457		
Order Desulfovibrionales	FEV1	13	MR Egger	0.025	0.027	0.386	0.608	0.982
			Weighted median	0.022	0.014	0.115		
			Inverse variance weighted	0.025	0.010	0.011		
			Weighted mode	0.018	0.019	0.348		
Family Desulfovibrionaceae	FEV1	12	MR Egger	0.027	0.028	0.360	0.532	0.930
			Weighted median	0.020	0.015	0.177		
			Inverse variance weighted	0.024	0.010	0.018		
			Weighted mode	0.014	0.019	0.482		
Family Lachnospiraceae	FEV1	18	MR Egger	-0.008	0.023	0.747	0.591	0.579
			Weighted median	-0.014	0.013	0.294		
			Inverse variance weighted	-0.019	0.009	0.035		
			Weighted mode	-0.003	0.021	0.871		
Genus Butyricimonas	FEV1	18	MR Egger	-0.037	0.036	0.324	0.041	0.665
			Weighted median	-0.016	0.011	0.145		
			Inverse variance weighted	-0.021	0.010	0.026		
			Weighted mode	-0.006	0.019	0.752		
Genus FamilyXIIIAD3011group	FEV1	15	MR Egger	0.029	0.099	0.773	<0.001	0.800
			Weighted median	0.043	0.014	0.003		
			Inverse variance weighted	0.054	0.020	0.007		
			Weighted mode	0.045	0.021	0.050		
Genus Oscillospira	FEV1	9	MR Egger	-0.017	0.043	0.707	0.544	0.875
			Weighted median	-0.021	0.014	0.116		
			Inverse variance weighted	-0.024	0.010	0.019		
			Weighted mode	-0.021	0.021	0.340		
Genus Parasutterella	FEV1	17	MR Egger	-0.042	0.049	0.406	<0.001	0.840
			Weighted median	-0.007	0.013	0.572		
			Inverse variance weighted	-0.033	0.015	0.030		
			Weighted mode	0.001	0.020	0.963		
Genus Unknowngenus	FEV1	20	MR Egger	0.007	0.038	0.863	0.693	0.692
			Weighted median	0.017	0.011	0.125		
			Inverse variance weighted	0.022	0.008	0.006		
			Weighted mode	0.015	0.021	0.477		

**Table 4:** Associations of genetic predisposition to gut microbiome with the value of FVC.

Exposure	Outcome	n(SNP)	Method	β	*SE*	*P*	*P* for IVW Q statistic	*P* for MR-Egger intercept test
Class Verrucomicrobiae	FVC	13	MR Egger	0.014	0.051	0.790	0.009	0.770
			Weighted median	0.010	0.014	0.472		
			Inverse variance weighted	0.029	0.013	0.031		
			Weighted mode	0.001	0.018	0.970		
Order Verrucomicrobiales	FVC	13	MR Egger	0.014	0.051	0.790	0.009	0.770
			Weighted median	0.010	0.014	0.465		
			Inverse variance weighted	0.029	0.013	0.031		
			Weighted mode	0.001	0.018	0.970		
Family Verrucomicrobiaceae	FVC	13	MR Egger	0.014	0.051	0.791	0.009	0.769
			Weighted median	0.010	0.014	0.456		
			Inverse variance weighted	0.029	0.013	0.032		
			Weighted mode	0.001	0.020	0.974		
Genus Akkermansia	FVC	13	MR Egger	0.014	0.051	0.788	0.009	0.771
			Weighted median	0.010	0.014	0.487		
			Inverse variance weighted	0.029	0.013	0.031		
			Weighted mode	0.001	0.018	0.967		
Genus Eubacteriumnodatumgroup	FVC	11	MR Egger	0.043	0.024	0.106	0.859	0.246
			Weighted median	0.012	0.007	0.085		
			Inverse variance weighted	0.014	0.005	0.010		
			Weighted mode	0.013	0.012	0.291		
Genus Fusicatenibacter	FVC	20	MR Egger	0.000	0.057	1.000	0.001	0.543
			Weighted median	0.031	0.014	0.024		
			Inverse variance weighted	0.034	0.014	0.015		
			Weighted mode	0.039	0.022	0.095		
Genus Lachnospira	FVC	7	MR Egger	-0.172	0.103	0.157	0.151	0.290
			Weighted median	-0.025	0.021	0.225		
			Inverse variance weighted	-0.052	0.019	0.007		
			Weighted mode	-0.020	0.026	0.466		
Genus Oscillospira	FVC	9	MR Egger	-0.011	0.058	0.860	0.134	0.769
			Weighted median	-0.020	0.015	0.175		
			Inverse variance weighted	-0.028	0.013	0.030		
			Weighted mode	-0.017	0.021	0.442		
Genus Parasutterella	FVC	17	MR Egger	-0.043	0.042	0.317	<0.001	0.755
			Weighted median	-0.010	0.013	0.425		
			Inverse variance weighted	-0.031	0.013	0.016		
			Weighted mode	-0.004	0.025	0.861		
Genus RuminococcaceaeUCG014	FVC	18	MR Egger	0.014	0.033	0.664	0.067	0.712
			Weighted median	0.007	0.013	0.575		
			Inverse variance weighted	0.026	0.011	0.015		
			Weighted mode	0.002	0.017	0.910		

**Table 5:** Associations of genetic predisposition to gut microbiome with the value of FEV1/FVC.

Exposure	Outcome	n(SNP)	Method	β	*SE*	*P*	*P* for IVW Q statistic	*P* for MR-Egger intercept test
Class Deltaproteobacteria	FEV1/FVC	14	MR Egger	0.014	0.036	0.703	0.107	0.753
			Weighted median	0.020	0.014	0.162		
			Inverse variance weighted	0.025	0.012	0.037		
			Weighted mode	0.021	0.020	0.326		
Order Desulfovibrionales	FEV1/FVC	13	MR Egger	0.024	0.033	0.484	0.213	0.980
			Weighted median	0.018	0.014	0.211		
			Inverse variance weighted	0.024	0.011	0.031		
			Weighted mode	0.019	0.019	0.340		
Family Desulfovibrionaceae	FEV1/FVC	12	MR Egger	0.020	0.034	0.566	0.174	0.859
			Weighted median	0.020	0.015	0.177		
			Inverse variance weighted	0.026	0.012	0.031		
			Weighted mode	0.020	0.019	0.303		
Family FamilyXIII	FEV1/FVC	14	MR Egger	0.014	0.049	0.773	0.079	0.810
			Weighted median	0.026	0.016	0.094		
			Inverse variance weighted	0.026	0.013	0.049		
			Weighted mode	0.043	0.030	0.174		
Genus Ruminococcus2	FEV1/FVC	15	MR Egger	0.011	0.032	0.722	0.117	0.686
			Weighted median	0.019	0.013	0.134		
			Inverse variance weighted	0.024	0.011	0.037		
			Weighted mode	0.019	0.019	0.344		
Genus Terrisporobacter	FEV1/FVC	6	MR Egger	-0.051	0.029	0.154	0.677	0.337
			Weighted median	-0.016	0.013	0.218		
			Inverse variance weighted	-0.021	0.010	0.035		
			Weighted mode	-0.010	0.021	0.637		
Genus Unknowngenus	FEV1/FVC	11	MR Egger	0.007	0.031	0.833	0.753	0.513
			Weighted median	0.028	0.013	0.027		
			Inverse variance weighted	0.027	0.009	0.004		
			Weighted mode	0.028	0.018	0.150		

**Table 6:** Genetic associations between lung function and gut microbiome.

Exposure	Outcome	n(SNP)	Method	β	*SE*	*P*	*P* for IVW Q statistic	*P* for MR-Egger intercept test
FEV1	Phylum Actinobacteria	4	MR Egger	3.325	3.273	0.417	0.289	0.938
			Weighted median	3.904	0.457	<0.001		
			Inverse variance weighted	3.611	0.359	<0.001		
			Weighted mode	4.043	0.790	0.014		
FEV1	Class Actinobacteria	4	MR Egger	6.726	5.475	0.344	0.016	0.728
			Weighted median	4.674	0.603	<0.001		
			Inverse variance weighted	4.554	0.623	<0.001		
			Weighted mode	5.240	1.343	0.030		
FEV1	Order Bifidobacteriales	4	MR Egger	6.374	4.966	0.328	0.058	0.781
			Weighted median	5.024	0.595	<0.001		
			Inverse variance weighted	4.812	0.558	<0.001		
			Weighted mode	5.385	1.088	0.016		
FEV1	Family Bifidobacteriaceae	4	MR Egger	6.374	4.966	0.328	0.058	0.781
			Weighted median	5.024	0.580	<0.001		
			Inverse variance weighted	4.812	0.558	<0.001		
			Weighted mode	5.385	1.133	0.018		
FEV1	Genus Bifidobacterium	4	MR Egger	6.008	4.863	0.342	0.074	0.843
			Weighted median	5.202	0.588	<0.001		
			Inverse variance weighted	4.923	0.540	0.000		
			Weighted mode	5.381	1.165	0.019		
FEV1	Genus Ruminiclostridium9	2	Inverse variance weighted	2.445	0.426	<0.001	0.271	NA
FVC	Phylum Actinobacteria	6	MR Egger	5.877	1.957	0.040	0.333	0.237
			Weighted median	3.275	0.341	<0.001		
			Inverse variance weighted	3.179	0.248	<0.001		
			Weighted mode	3.569	0.580	0.002		
FVC	Class Actinobacteria	7	MR Egger	15.138	4.901	0.027	<0.001	0.063
			Weighted median	3.707	0.436	<0.001		
			Inverse variance weighted	3.585	0.884	<0.001		
			Weighted mode	4.164	0.562	<0.001		
FVC	Order Bifidobacteriales	7	MR Egger	15.453	5.375	0.035	<0.001	0.079
			Weighted median	4.119	0.446	<0.001		
			Inverse variance weighted	3.742	0.931	<0.001		
			Weighted mode	4.462	0.564	<0.001		
FVC	Family Bifidobacteriaceae	7	MR Egger	15.453	5.375	0.035	<0.001	0.079
			Weighted median	4.119	0.450	<0.001		
			Inverse variance weighted	3.742	0.931	<0.001		
			Weighted mode	4.462	0.567	<0.001		
FVC	Genus Bifidobacterium	7	MR Egger	15.372	5.402	0.036	<0.001	0.084
			Weighted median	4.282	0.448	<0.001		
			Inverse variance weighted	3.843	0.927	<0.001		
			Weighted mode	4.492	0.563	<0.001		
FVC	Genus Ruminiclostridium9	2	Inverse variance weighted	2.328	0.336	<0.001	0.695	NA

NA, not applicable.

### 3.2 Causal effect of gut microbiota on IPF

**Figure 2:**
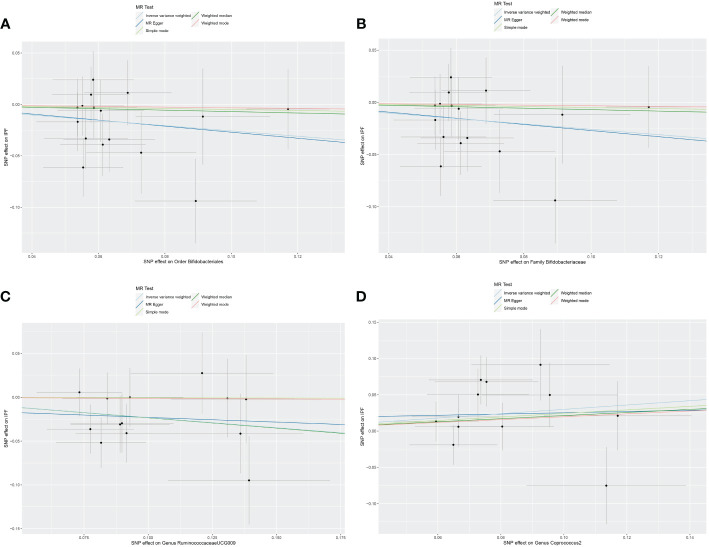
Scatter plot of the causal effect of gut microbiota on IPF. **(A)** Scatter plot for the causal effect of *Order Bifidobacteriales* on IPF risk. **(B)** Scatter plot for the causal effect of *Family Bifidobacteriaceae* on IPF risk. **(C)** Scatter plot for the causal effect of *Genus RuminococcaceaeUCG009* on IPF risk. **(D)** Scatter plot for the causal effect of *Genus Coprococcus2* on IPF risk.

The original GWAS involving 18,340 individuals from 24 cohorts provided summary statistics for 211 microbial taxa. Estimated by the IVW test, four taxa were identified. The MR analysis revealed that the abundance of *Order Bifidobacteriales* (OR=0.773, 95% CI: 0.610–0.979, *p*=0.033), *Family Bifidobacteriaceae* (OR=0.773, 95% CI: 0.610–0.979, *p*=0.033), and *Genus RuminococcaceaeUCG009* (OR=0.793, 95% CI: 0.652–0.965, *p*=0.020) have a protective effect against IPF. Increased abundance of *Genus Coprococcus2* was associated with a higher risk of IPF (OR=1.349, 95% CI: 1.021–1.783, *p*=0.035). Subsequently, Cochrane’s Q test revealed that there was heterogeneity (*p*<0.05). MR-PRESSO test and MR-Egger intercept tests identified no pleiotropy or significant outliers (*p*>0.05). The causal effect between 211 microbial taxa and IPF was presented in **Table 2**. The scatterplot was shown in **Figure 2**. All IVs used in our study were provided in **Supplementary Table S2**.

### 3.3 Causal effect of gut microbiota on FEV_1_

**Figure 3:**
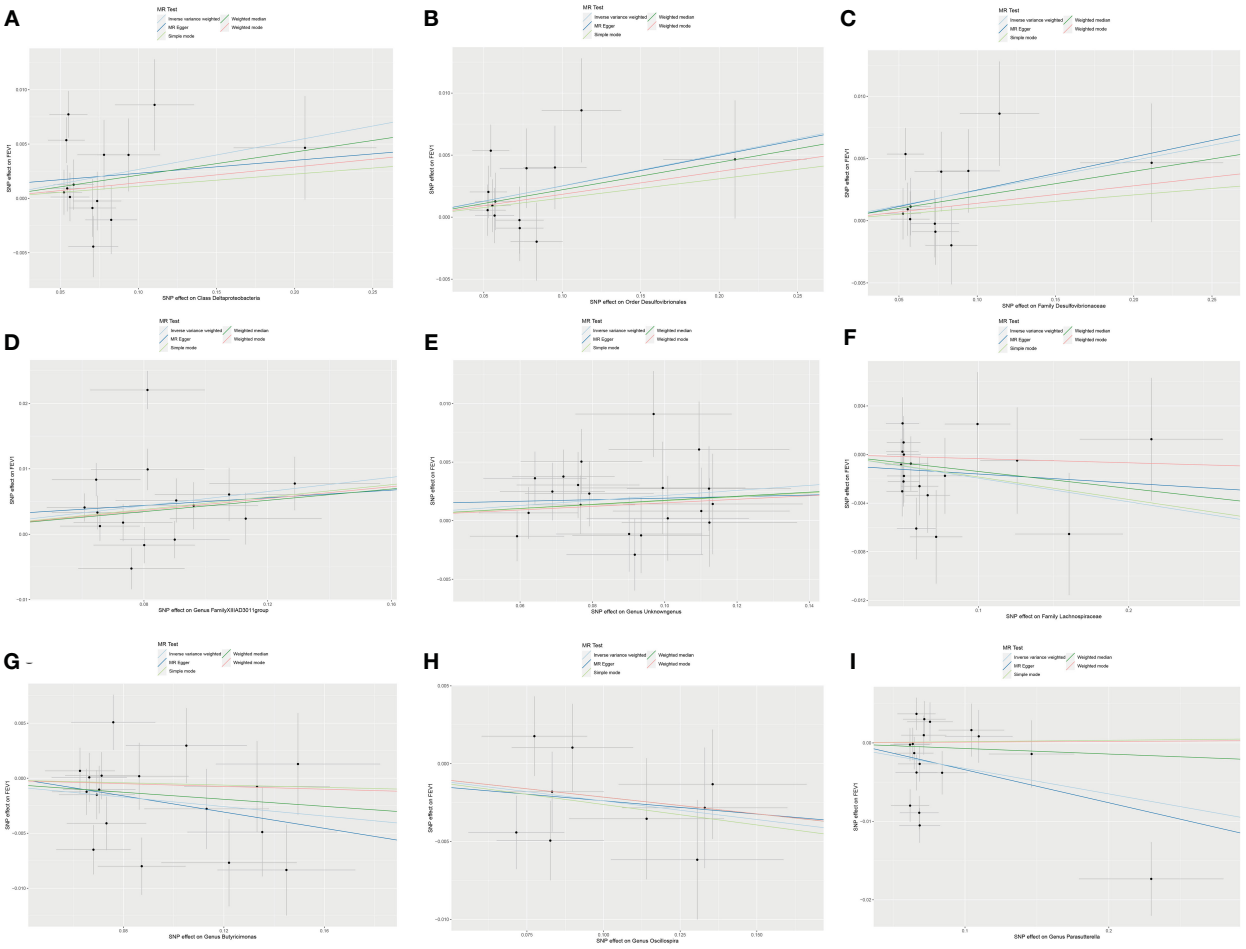
Scatter plot of the causal effect of gut microbiota on FEV_1_. **(A)** Scatter plot for the causal effect of *Class Deltaproteobacteria* on FEV_1_. **(B)** Scatter plot for the causal effect of *Order Desulfovibrionales* on FEV_1_. **(C)** Scatter plot for the causal effect of *Family Desulfovibrionaceae* on FEV_1_. **(D)** Scatter plot for the causal effect of *Genus FamilyXIIIAD3011group* on FEV_1_. **(E)** Scatter plot for the causal effect of *Genus Unknowngenus* on FEV_1_. **(F)** Scatter plot for the causal effect of *Family Lachnospiraceae* on FEV_1_. **(G)** Scatter plot for the causal effect of *Genus Butyricimonas* on FEV_1_. **(H)** Scatter plot for the causal effect of *Genus Oscillospira* on FEV_1_. **(I)** Scatter plot for the causal effect of *Genus Parasutterella* on FEV_1_.

Nine causal relationships were identified between the gut microbiota and FEV_1_ (**Table 3**). The elevation abundance of *Class Deltaproteobacteria* (β=0.027, se=0.013, *p*=0.042), *Order Desulfovibrionales* (β=0.025, se=0.010, *p*=0.011), *Family Desulfovibrionaceae* (β=0.024, se=0.010, *p*=0.018), *Genus FamilyXIIIAD3011group* (β=0.054, se=0.020, *p*=0.007), and *Genus Unknowngenus* (β=0.022, se=0.008, *p*=0.006) were associated with the raise of FEV_1_. However, *Family Lachnospiraceae* (β=-0.019, se=0.009, *p*=0.035), *Genus Butyricimonas* (β=-0.021, se=0.010, *p*=0.026), *Genus Oscillospira* (β=-0.024, se=0.010, *p*=0.019), and *Genus Parasutterella* (β=-0.033, se=0.015, *p*=0.030) were associated with impairment of FEV_1_. The results of Cochran’s Q test showed that obvious heterogeneity was found in the selected SNPs of *Genus FamilyXIIIAD3011group, Genus Parasutterella, Class Deltaproteobacteria*, and *Genus Butyricimonas* (*p*<0.05). No overall horizontal pleiotropy existed in any of the IVs, as shown by the results of the MR-Egger intercept test (*p*>0.05). Finally, the leave-one-out method achieved stable results after excluding the SNP one by one. **Table 3** listed the associations of genetic predisposition to gut microbiome with the value of FEV_1_. The scatterplot was shown in **Figure 3**.

### 3.4 Causal effect of gut microbiota on FVC

As set out in **Table 4**. ten causal relationships were identified between the gut microbiota and FVC. The abundance of *Class Verrucomicrobiae* (β=0.029, se=0.013, *p*=0.031), *Order Verrucomicrobiales* (β=0.029, se=0.013, *p*=0.031), *Family Verrucomicrobiaceae* (β=0.029, se=0.013, *p*=0.031), *Genus Akkermansia* (β=0.029, se=0.013, *p*=0.031), *Genus Eubacteriumnodatumgroup* (β=0.014, se=0.005, *p*=0.010), *Genus Fusicatenibacter* (β=0.034, se=0.014, *p*=0.015), and *Genus RuminococcaceaeUCG014* (β=0.026, se=0.011, *p*=0.015) were associated with the improvement of FVC. *Genus Lachnospira* (β=-0.052, se=0.019, *p*=0.007), *Genus Oscillospira* (β=-0.028, se=0.013, *p*=0.030), and *Genus Parasutterella* (β=-0.031, se=0.013, *p*=0.016) were associated with the reduction of FVC. To further evaluate the results, heterogeneity analyses were conducted. Results from Cochrane’s Q test showed that there was heterogeneity found in the selected SNPs among the data of *Genus Akkermansia, Family Verrucomicrobiaceae, Genus Parasutterella, Class Verrucomicrobiae, Order Verrucomicrobiales*, and *Genus Fusicatenibacter* (*p*<0.05). The MR-Egger intercept tests showed that there is no pleiotropy or outliers (*p*>0.05), suggesting that the IVs are unlikely to affect FVC through pathways other than the mentioned indicators.

### 3.5 Causal effect of gut microbiota on FEV_1_/FVC

**Figure 4:**
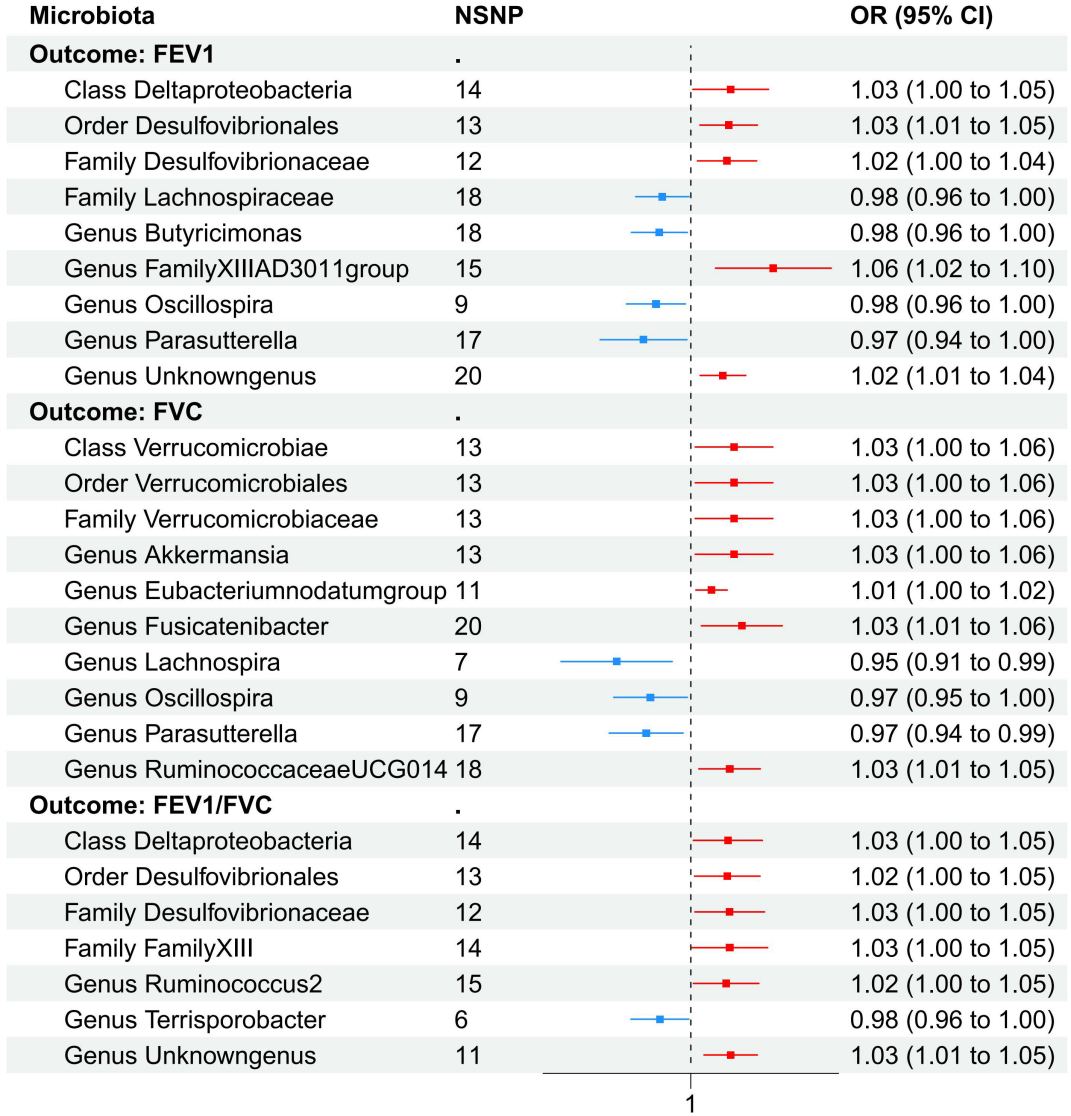
The forest plot illustrated all exposure factors related to pulmonary function identified by two-sample MR analysis

As shown in **Table 5**, *Class Deltaproteobacteria* (β=0.025, se=0.012, *p*=0.037), *Order Desulfovibrionales* (β=0.024, se=0.011, *p*=0.031), *Family Desulfovibrionaceae* (β=0.026, se=0.012, *p*=0.031), *Family FamilyXIII* (β=0.026, se=0.013, *p*=0.049), *Genus Ruminococcus2* (β=0.024, se=0.011, *p*=0.037), and *Genus unknowngenus* (β=0.027, se=0.009, *p*=0.004) demonstrated positive associations with the increase of FEV_1_/FVC. *Genus Terrisporobacter* (β=-0.021, se=0.010, *p*=0.035) had a negative correlation with FEV_1_/FVC. Several sensitivity tests were conducted for additional confirmation of the robustness of the results. All results of Cochran’s Q test indicated that there was no significant heterogeneity (*p*>0.05). Moreover, the MR-Egger intercept test and the global test p-values both revealed no statistically significant results (*p*>0.05), suggesting no presence of horizontal pleiotropy. The leave-one-out sensitivity analysis showed stable results of the effect of *Genus unknowngenus* on FEV_1_/FVC. The forest plot illustrated all exposure factors related to pulmonary function indices (including FEV1,FVC, and FEV1/FVC) (**Figure 4**).

### 3.6 Causal effect of lung function on gut microbiota

To understand the consequences of IPF and lung function on the abundance of the gut microbiome, reverse two-sample MR tests were performed. Due to the insufficient number of analyzable SNPs when considering IPF and FEV_1_/FVC as exposure factors, further MR analysis and subsequent heterogeneity analyses cannot be conducted. Therefore, the reverse MR analysis in this study only focuses on the casual effects of FEV_1_ and FVC on the abundance of gut microbiota.

The increase of FEV_1_ can amplify the abundance of *Phylum Actinobacteria* (β=3.611, se=0.359, *p*<0.001), *Class Actinobacteria* (β=4.554, se=0.623 *p*<0.001), *Order Bifidobacteriales* (β=4.812, se=0.558, *p*<0.001), *Family Bifidobacteriaceae* (β=4.812, se=0.558, *p*<0.001), *Genus Bifidobacterium* (β=4.923, se=0.540, *p*<0.001), and *Genus Ruminiclostridium9* (β=2.445, se=0.426, *p*<0.001). When investigating the impact of FVC on gut microbiota, a promoting trend for the abundance of the same gut microbiota was observed. Detailed significant results for the causal relationships are listed in **Table 6**. None horizontal pleiotropy was detected at statistically significant levels (all *p* for MR-Egger intercept test > 0.05).

### 3.7 Fatty acids were identified as effective mediators by MVMR analysis

Considering the possible impact of fatty acids on the progression from specific gut microbiota to IPF, MVMR analysis was conducted by adjusting for significant associations with fifteen indicators of fatty acids. Drawing on earlier research findings of this study, we hypothesized that the enhancement of FEV_1_ could lead to increased abundance of *Bifidobacteriales*, resulting in a reduced risk of IPF.

**Figure 5:**
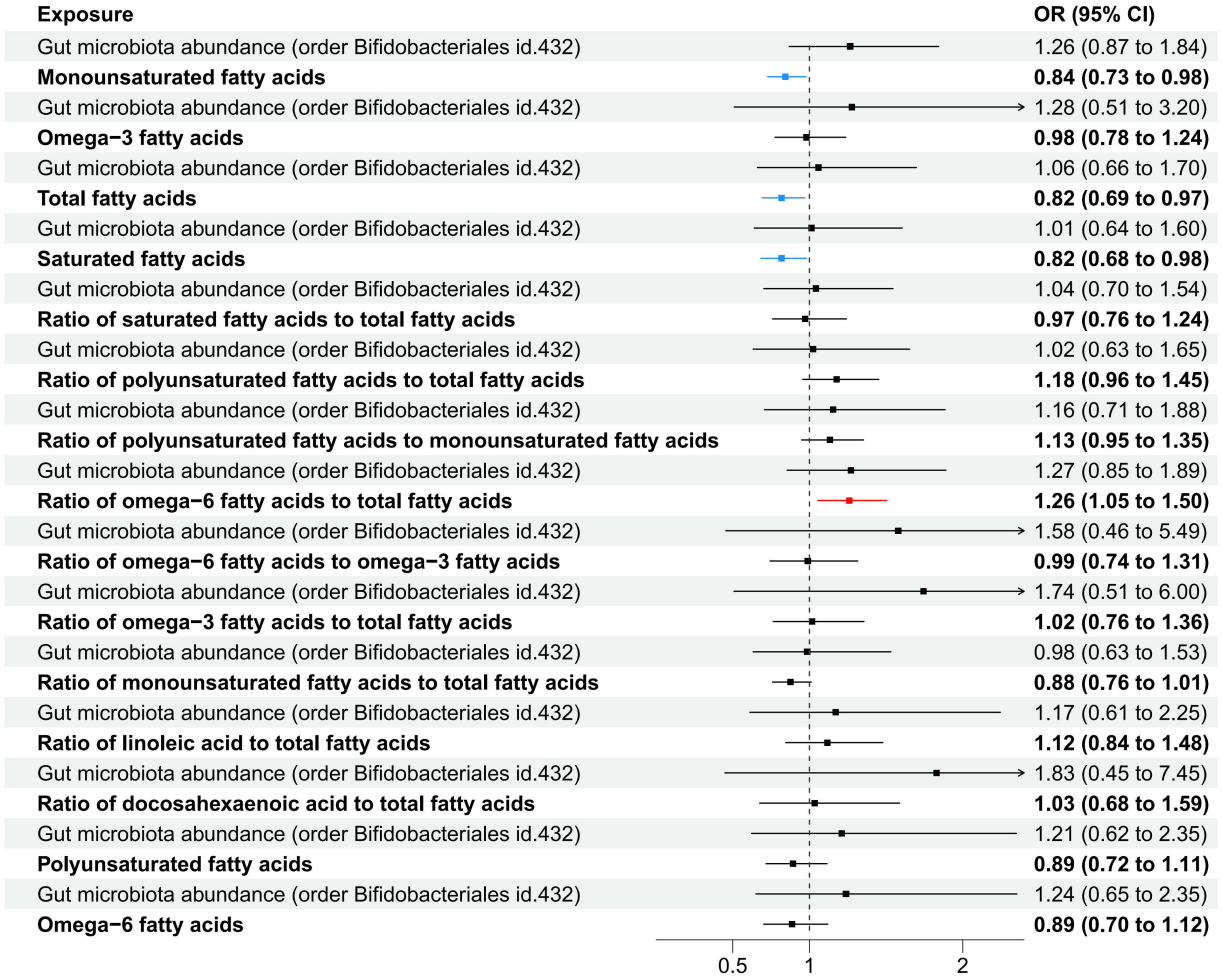
A forest plot displayed the factors related to fatty acids identified through MVMR analysis, with Order Bifidobacteriales as the exposure and IPF as the outcome variable.

In the MVMR analysis, our focus was on the abundance of gut microbiota of the *Bifidobacteriales order*. Adjustments were made for one fatty acid at a time, with *Bifidobacteriales* serving as a co-exposure, to assess their potential mediation effects on IPF. The findings, as presented in **Table 7**. Four factors related to fatty acids showed potential mediation in linking *Bifidobacteriales* and IPF: monounsaturated fatty acids (OR=0.84, 95% CI: 0.73–0.98, *p*=0.024), total fatty acids (OR=0.82, 95% CI: 0.69–0.97, *p*=0.021), saturated fatty acids (OR=0.82, 95% CI: 0.68–0.98, *p*=0.030), and the ratio of omega-6 fatty acids to total fatty acids (OR=1.26, 95% CI: 1.05–1.50, *p*=0.011). The forest plot depicting these results is presented in **Figure 5**.

**Table 7:** MVMR results of causal relationships between gut microbiota abundance (order Bifidobacteriales) and IPF after adjusting for fatty acids.

Exposure	Outcome	n(SNP)	OR (95% CI)	*P*
order Bifidobacteriales	IPF	1	1.26 (0.87–1.84)	0.221
Monounsaturated fatty acids	IPF	45	0.84 (0.73–0.98)	0.024
order Bifidobacteriales	IPF	1	1.28 (0.51–3.20)	0.601
Omega-3 fatty acids	IPF	38	0.98 (0.78–1.24)	0.865
order Bifidobacteriales	IPF	1	1.06 (0.66–1.70)	0.811
Total fatty acids	IPF	48	0.82 (0.69–0.97)	0.021
order Bifidobacteriales	IPF	1	1.01 (0.64–1.60)	0.953
Saturated fatty acids	IPF	40	0.82 (0.68–0.98)	0.030
order Bifidobacteriales	IPF	1	1.04 (0.70–1.54)	0.836
Ratio of saturated fatty acids-total fatty acids	IPF	18	0.97 (0.76–1.24)	0.817
order Bifidobacteriales	IPF	1	1.02 (0.63–1.65)	0.925
Ratio of polyunsaturated fatty acids-total fatty acids	IPF	31	1.18 (0.96–1.45)	0.124
order Bifidobacteriales	IPF	1	1.16 (0.71–1.88)	0.565
Ratio of polyunsaturated fatty acids-monounsaturated fatty acids	IPF	39	1.13 (0.95–1.35)	0.166
order Bifidobacteriales	IPF	1	1.27 (0.85–1.89)	0.237
Ratio of omega-6 fatty acids-total fatty acids	IPF	34	1.26 (1.05–1.50)	0.011
order Bifidobacteriales	IPF	1	1.58 (0.46–5.49)	0.472
Ratio of omega-6 fatty acids-omega-3 fatty acids	IPF	25	0.99 (0.74–1.31)	0.941
order Bifidobacteriales	IPF	1	1.74 (0.51–6.00)	0.377
Ratio of omega-3 fatty acids-total fatty acids	IPF	24	1.02 (0.76–1.36)	0.903
order Bifidobacteriales	IPF	1	0.98 (0.63–1.53)	0.946
Ratio of monounsaturated fatty acids-total fatty acids	IPF	40	0.88 (0.76–1.01)	0.072
order Bifidobacteriales	IPF	1	1.17 (0.61–2.25)	0.631
Ratio of linoleic acid-total fatty acids	IPF	25	1.12 (0.84–1.48)	0.436
order Bifidobacteriales	IPF	1	1.83 (0.45–7.45)	0.399
Ratio of docosahexaenoic acid-total fatty acids	IPF	20	1.03 (0.68–1.59)	0.874
order Bifidobacteriales	IPF	1	1.21 (0.62–2.35)	0.574
Polyunsaturated fatty acids	IPF	42	0.89 (0.72–1.11)	0.320
order Bifidobacteriales	IPF	1	1.24 (0.65–2.35)	0.515
Omega-6 fatty acids	IPF	40	0.89 (0.70–1.12)	0.313

## 4 Discussion

Using the summary statistics of gut microbiota from the largest GWAS meta-analysis conducted by the MiBioGen consortium (Kurilshikov et al., 2021) and the latest summary statistics of IPF (Allen et al., 2022), FEV_1_, FVC and FEV_1_/FVC (Shrine et al., 2023) limited to European ancestry, we performed a bidirectional two-sample MR analysis to evaluate the causal association between gut microbiota and IPF and lung function. Four taxa were found causally associated with the risk of IPF. *Order Bifidobacteriales, Family Bifidobacteriaceae*, and *Genus RuminococcaceaeUCG009* exerted protective effects on IPF, while *Genus Coprococcus2* promote the development of IPF. Several taxa were causally associated with lung function. Among them, the most prominent beneficial microbiota comprised by *Class Deltaproteobacteria, Order Desulfovibrionales, Family Desulfovibrionaceae*, *Class Verrucomicrobiae, Order Verrucomicrobiales*, and *Family Verrucomicrobiaceae*. Meanwhile, *Family Lachnospiraceae, Genus Oscillospira*, and *Genus Parasutterella* were associated with the impairment of lung function. In the reverse MR analysis, the abundance of *Order Bifidobacteriales, Family Bifidobacteriaceae*, and *Genus Bifidobacterium* increased with the improvement of FEV_1_ and FVC. The MVMR results suggested that fatty acids (monounsaturated fatty acids, total fatty acids, saturated fatty acids, and ratio of omega-6 fatty acids to total fatty acids) probably played a role in the genetic pathway from the gut microbiota to IPF, especially for *Bifidobacteriales*.

The lung is the largest human organ in direct contact with the environment. The lung was considered non-sterile partly due to the failure of isolating bacteria in lung specimens with traditional culture techniques. Only in the setting of infections, such as pneumonia or bronchiectasis, as in such disorders microbes could be culture-isolated and considered pathogenic of the disease (Ntolios et al., 2021). The introduction of sequencing of the 16S rRNA gene technique allowed recognition of the fact that bacteria not only exist within the human lung but are altered in lung disease and correlate with alveolar immunity and clinical outcomes (Invernizzi et al., 2020). The normal lung microbiota is primarily composed of gram-negative bacteria and facultative anaerobes. A previous MR analysis has suggested that gut microbiota can impact chronic respiratory diseases (CRDs), including chronic obstructive pulmonary disease (COPD), asthma, interstitial lung disease (ILD), sarcoidosis and occupational lung diseases (Shi et al., 2023). The presence and abundance of specific gut microbiota in various disease conditions imply a potential role in modulating immune responses and contributing to the development or resolution of inflammation-related disorders.

Utilizing 16S rRNA gene sequencing, Wei’s research (Wei et al., 2023) indicates that bleomycin (BLM) induced pulmonary fibrosis (PF) could alter the relative abundance of many microbiotas in mice gut. At the family level, compared to the control group, the abundance of *Bifidobacteriaceae, Erysipelotrichaceae*, and *Lactobacillaceae* showed an elevation in PF group, while some beneficial microbiota was significantly decreased, such as *Bacilaceae* and *Lachnospiraceae*. At the genus level, the abundance of *Akkermansia, Bacillus*, and *Lactobacillus* showed reduction in the PF group while was significantly increased for *Clostridium, Erysipelatoclostridium, Faecalibaculum*, and *Lachnoclostridium* compared to the control group. Consistent with Wei’s study, we used MR to validate the genetic beneficial effect of Genus Akkermansia on lung function. Recent investigations have elucidated the significance of Akkermansia muciniphila (A. muciniphila) in modulating the pathophysiology of interstitial lung diseases, notably in cystic fibrosis (CF) and COVID-19 convalescent patients. Pharmacological interventions aimed at augmenting A. muciniphila populations have demonstrated the potential to alleviate intestinal inflammation (Manor et al., 2016), diminish pathogenic bacterial loads, and thereby emerge as a therapeutic target for microbiome-based therapies in CF. According to a study performed on SARS-CoV-2 recovered patients (Yeoh et al., 2021), an elevated presence of A. muciniphila correlates with markers of inflammation, implicating its association with dysbiosis of the gut microbiota and systemic inflammatory responses during disease states. Yoon and colleagues (Yoon et al., 2021) extracted genomic DNA from lung tissues of patients with IPF and found that the relative abundance of *Lactobacillus, Paracoccus*, and *Akkermansia* was increased in patients with IPF compared with that in the controls. Based on the findings of our study, which suggest that *Akkermansia* acts as a protective factor for lung function, it is warranted for further investigation of the specific mechanisms of *Akkermansia* for the impact on IPF. Wei et.al (Wei et al., 2023) also concluded that *Family Bifidobacteriaceae* would exacerbate the microbial burden in IPF and promote disease progression, while our findings suggested that an increase in FEV_1_ can give rise to the abundance of *Family Bifidobacteriaceae*. The latter was present as a probiotic to reduce the risk of IPF.A study (Yeoh et al., 2021) conducted on individuals who recovered from SARS-CoV-2 also observed that certain species of *Bifidobacteria* were present at reduced levels.

Quan and colleagues (Quan et al., 2022) showed that after BLM induced PF in mice, the microecological balance of the gut microbiota was destroyed, and the relative abundance of some intestinal probiotics like *Firmicutes, Lactobacillales, Lactobacillaceae, Lactobacillus*, and *Catenibacterium* dramatically lowered while the relative abundance of *Verrucomicrobiales* and *Enterobacteriales* remarkably increased. Differently, our study found that *Family Lachnospiraceae* was associated with impairment of FEV_1_. Yoon’s study (Yoon et al., 2021) revealed a relatively increased abundance of *Lactobacillus* and *Bifidobacterium* in the lung tissue of IPF patients. *Lactobacillus* generally resides in the gastrointestinal and reproductive tract, where it maintains a healthy microecology with lactic acid production. However, given the well-known association between IPF and gastroesophageal reflux disease (Cheng et al., 2023), the high prevalence of GERD in IPF might contribute to the increase in the relative abundance of *Lactobacillus* in IPF. Levels of lactic acid and lactate dehydrogenase-5, which induce the differentiation of fibroblasts into myofibroblasts by activating transforming growth factor (TGF)-ß1, were elevated in lung tissues from patients with IPF compared with healthy persons (Kottmann et al., 2012). Therefore, bacteria that produce lactic acid might also contribute to the progression of IPF. However, a different conclusion drawn by wang and colleagues (Wang et al., 2022) was that Lactobacillus mucosae can regulate immune responses and intestinal micro-ecological balance by reducing the proportions of inflammatory cells, including granulocytes and monocytes in the blood, and increasing interferon (IFN)-β, interleukin (IL)-1β, IL-10, and tumor necrosis factor (TNF)-α levels. The observed discrepancies in the role of lactic acid-producing bacteria, notably Lactobacillus mucosae, concerning IPF progression, may be attributed to multifactorial influences and intricate mechanisms. Firstly, strain-specific variations play a pivotal role, as different strains within the same species exhibit diverse immunomodulatory capacities, thereby influencing their impact on IPF. Secondly, individual host characteristics, encompassing genetic predispositions, immune status, and general health conditions, exert a substantial effect on the host’s response to bacterial interventions. This inherent variability among IPF patients might account for the divergent outcomes following exposure to Lactobacillus mucosae. Lastly, the efficacy of Lactobacillus mucosae seems to be intricately tied to the administered dose and duration of treatment, thereby accentuating the necessity for meticulous attention to these variables in forthcoming research endeavors.

This MR study found that an increase in *Genus Parasutterella* abundance leads to a decrease in FEV_1_ and FVC. Gong and colleagues (Gong et al., 2021) calculated the sequence proportions of microbiome and made comparison between fibrotic animals and control ones, showing that *Parasutterella* were synchronously up-regulated in PF group and Parasutterella was negatively correlated with thymidine. There are very close correlations between distinctive gut microbiota and metabolites under pulmonary fibrotic pathological conditions (Bai et al., 2019; Fang et al., 2020). In recent scientific explorations centered around the “gut-lung axis,” alterations in gut microbial composition and their metabolic byproducts have been implicated in the development of fibrotic interstitial lung diseases. The gut microbiome participates in lung fibrogenesis through metabolically mediated pathways. TGF-β-driven stimulation of fibroblasts enhances glutamine and glutamate concentrations, necessitating glutaminolysis for myofibroblast differentiation and activation (Bernard et al., 2018). In macrophages, arginine boosts glutathione levels and curbs the secretion of pro-inflammatory cytokines such as TNF-α, IL-1β, and IL-6, with arginine derivatives like iNOS and cNOS exerting bidirectional effects on airway inflammation (Fu et al., 2020; Wu et al., 2021). Arginine also restrains NF-κB activation and suppresses MMP-2 and MMP-9 activities implicated in fibrosis (Hnia et al., 2008). Tryptophan, interacting with aryl hydrocarbon receptors, dampens pro-inflammatory T-cell subsets (Takei et al., 2020), and upon conversion to 5-MTP in fibroblasts, it impedes macrophage activation and inflammatory mediator release by interfering with TGF-β/SMAD3 and PI3K/Akt signaling, concurrently hindering myofibroblast formation (Fang et al., 2020). Furthermore, butyrate inhibits TGF-β and fibroblast expression through HDAC-mediated histone acetylation (Park et al., 2021), whereas bile acids stimulate the TGF-β1/Smad3 pathway, promoting alveolar epithelial and lung fibroblast activation (Chen et al., 2017). Collectively, these findings underscore the complex interplay between gut-derived metabolites and the progression of lung fibrosis, highlighting potential therapeutic avenues.

Our study suggested the potential protective roles of specific monounsaturated fatty acids and saturated fatty acids in the genetic association between *Bifidobacterium* and IPF. Previous research demonstrated that exposure to a high-fat diet rich in palmitic acid, a saturated fatty acid, increased lung fibrosis in wild-type mice after being administered bleomycin. This effect was found to be associated with the activation of the unfolded protein response and apoptosis of lung epithelial cells (Chu et al., 2019), suggesting that high intake of saturated fatty acids may be a contributing factor to the pathogenesis of EMT due to a defect in long-chain fatty acid family member 6 enzyme. Additionally, intratracheal administration of fatty acids has shown potential for therapeutic application in the treatment of PF (Zhao et al., 2014). These findings suggest that a diet rich in essential fatty acids or targeted delivery of fatty acids directly to the lungs may represent a promising approach for the prevention and treatment of lung fibrosis.

To our knowledge, this study is the first comprehensive investigation utilizing large-scale MR analysis to examine the causal relationship between gut microbiota, fatty acids, IPF, and lung function. We utilized the latest and largest GWAS data of individuals with European ancestry. The strength of the study lies in the use of MR analysis, which reduces the impact of measurement errors and addresses potential issues such as reverse causation and confounding factors commonly associated with observational studies. Furthermore, we conducted various sensitivity analyses using multiple complementary MR approaches to assess the robustness of the association and potential bias from pleiotropy. Overall, this study provides valuable insights into the gut-lung axis in IPF and contributes to the understanding of potential preventive and treatment strategies for this condition.

This study has limitations that need to be acknowledged. First, the study population is limited to individuals of European ancestry, thereby rendering the findings not generalizable to populations of other ancestral backgrounds. As IPF prevalence varies across regions and races, further research is warranted to determine regional or racial disparities. Second, heterogeneity among IPF patients may lead to inconsistent results, limiting the conclusions that can be drawn. The underlying mechanism of the lung-gut axis in IPF remains unclear as our research focuses on correlation analysis. Therefore, further investigations are needed to explore specific mechanisms. Comprehensive studies on the lung-gut axis are essential as factors such as seasonality, age, living habits, diet structure, genetic background, and treatment regimens may affect fecal composition in IPF patients. Such studies may provide new directions and strategies for the prevention and treatment of IPF.

## 5 Conclusion

In summary, the current study suggested the casual effects of the specific gut microbes on the risk of IPF and lung function. In turn, lung function also exerted a positive role in some gut microbes. Maintaining an appropriate dietary consumption of lipid substances can provide a certain level of protection against the development and progression of IPF. Our findings provided novel insights into the potential role of gut microbiota for IPF and indicated a potential mechanism of gut microbiota-mediated prevention of IPF.

## Data availability statement

The datasets presented in this study can be found in online repositories. The names of the repository/repositories and accession number(s) can be found in the article/**Supplementary Material**.

## Author contributions

YR: Writing – original draft, Software, Methodology, Investigation, Formal Analysis, Data curation, Conceptualization. YZ: Writing – review & editing, Methodology, Investigation, Data curation, Conceptualization. YC: Writing – review & editing, Methodology, Investigation, Data curation, Conceptualization. HQ: Writing – review & editing, Methodology, Investigation, Data curation, Conceptualization. HZ: Writing – review & editing, Supervision, Project administration, Funding acquisition.
