# Supplementary material for: Genetic liability of gut microbiota for idiopathic pulmonary fibrosis and lung function: a two-sample Mendelian randomization study
Source: Front Cell Infect Microbiol. 2024 May 22;14:1348685. doi: 10.3389/fcimb.2024.1348685 (PMC11150651; doi:10.3389/fcimb.2024.1348685)
Supplement: Supplementary file 1 [file DataSheet_1.zip › Supplement/Supplementary table S1.docx]

**Supplementary table 1.** **STROBE-MR checklist.**

| **Item No.** | **Section** | **Checklist item** | **Page No.** | **Relevant text from manuscript** |
| --- | --- | --- | --- | --- |
| 1 | **TITLE and ABSTRACT** | Indicate Mendelian randomization (MR) as the study’s design in the title and/or the abstract if that is a main purpose of the study | 1-2 | Genetic liability of gut microbiota for idiopathic pulmonary fibrosis and lung function: a two-sample Mendelian randomization study |
|  | **INTRODUCTION** |  |  |  |
| 2 | **Background** | Explain the scientific background and rationale for the reported study. What is the exposure? Is a potential causal relationship between exposure and outcome plausible? Justify why MR is a helpful method to address the study question | 1 | The microbiota-gut-brain axis has revealed a possible contribution of the gut microbiota to Idiopathic pulmonary fibrosis (IPF). However, population-level studies with a higher evidence level for the causality are lacking. |
| 3 | **Objectives** | State specific objectives clearly, including pre-specified causal hypotheses (if any). State that MR is a method that, under specific assumptions, intends to estimate causal effects | 1 | The objective of the two-sample Mendelian randomization (MR) analysis was to examine the casual relationship between gut microbiota and IPF and lung function. |
|  | **METHODS** |  | 5-11 |  |
| 4 | **Study design and data sources** | Present key elements of the study design early in the article. Consider including a table listing sources of data for all phases of the study. For each data source contributing to the analysis, describe the following: | 5-6 | See Figure 1 |
|  | a) | Setting: Describe the study design and the underlying population, if possible. Describe the setting, locations, and relevant dates, including periods of recruitment, exposure, follow-up, and data collection, when available. | 5 | See in Table 1. Study design overview and the assumptions of the Mendelian randomization (MR) study are shown in Figure 1. In MR research, the instrumental variable (IV) must meet three fundamental assumptions. Assumption i indicates that the genetic variants proposed as instrumental variables should be robustly associated with the risk factor of interest. Assumption ii indicates that the used genetic variants should not be associated with potential confounders. Assumption iii indicates that the selected genetic variants should affect the risk of the outcome merely through the risk factor, not via alternative pathways |
|  | b) | Participants: Give the eligibility criteria, and the sources and methods of selection of participants. Report the sample size, and whether any power or sample size calculations were carried out prior to the main analysis | 6-8 | The gut microbiota data used in this study were sourced from the largest genome-wide association study (GWAS) conducted by the MiBioGen consortium, which has curated and analyzed genome-wide genotypes and 16S fecal microbiome data, including 5,717,754 SNPs and 18,340 participants from 24 cohorts (total 211 taxa: 9 phylum, 16 classes, 20 orders, 35 families, and 131 genus)[15]. The GWAS data for fatty acids were extracted from MRC-IEU OpenGWAS project (https://gwas.mrcieu.ac.uk/). Detailed information was shown in Table 1. To ensure minimal overlap with IPF studies, we specifically selected studies with no or minimal sample overlap. After calculation, we obtained a maximum overlapping rate of <10%, which may not have been sufficient to affect our resultsThe data relevant to IPF were derived from the GWAS led by Richard J. Allen[21]. This study encompassed a cohort of 4125 cases and 20,464 controls, consisting of unrelated individuals of European ancestry from diverse regions including the USA, UK, and Spain. The diagnosis of IPF was conducted based on the guidelines established by the American Thoracic Society and the European Respiratory Society[22, 23] |
|  | c) | Describe measurement, quality control and selection of genetic variants | 9-11 | Instrument Selection and Data Harmonization  Given the limited number of available SNPs, we selected SNPs significantly related to the gut microbiota with a loose cutoff of p < 1e-5. Then, significant SNPs were clumped within 10,000 kb at the level of linkage disequilibrium (LD) r2 = 0.01 using the European reference panel of the 1000 Genome Project. In reverse MR analyses, independent SNPs were selected by LD (r2 < 0.01 within 250-kb clumping distance, based on the European reference panel of the 1000 Genome Project) at a compromised signifcant level (1e-5) due to the relatively insufficient variables.  2.5. Statistical analysis  The two-sample MR study was incorporated to evaluate the causal links between 211 microbial taxa and IPF and lung function. All SNPs were harmonized between the exposure and the outcome by alleles to ensure the alignment of effect. Steiger_filtering test was utilized to remove SNPs associated with reverse causality. IVW method was opted for the as the primary approach to assess the total causal effect of the exposure on the outcome. This involved minimizing the weighted average variance by combining 2 or more IVs, where each IV's weight was calculated as the inverse of the variance of the effect estimate[21]. In addition to the IVW method, we also conducted supplementary analyses, such as Egger regression (MR Egger), weighted median, and weighted mode. MR‒Egger allowed us to explore the average horizontal pleiotropic effect across IVs[22]. The weighted median will provide a consistent estimate if at least 50% of the weight comes from valid instruments and therefore is robust against instrumental outliers[23]. The weighted mode approach assumes that the most frequently occurring association estimate is unaffected by pleiotropy, meaning that it must correspond to the true causal effect[24]. The results of the casual effects are represented by β and Odds Ratio (OR) (OR = expβ). The proportions of trait variance explained by identified genetic instruments (R2) was estimated using the formula: R2 = (2β2 × EAF × (1-EAF))/(2β2 × EAF × (1-EAF) + 2 N × EAF × (1-EAF) × SE2), where EAF = effect allele frequency, β=effect size of the SNP in the exposure GWAS, SE = standard error, and N = sample size of the exposure GWAS[25, 26]. And the instrument strength was evaluated using the F statistic, where F = (R2 × (N-2))/(1-R2), to measure the statistical strength of each SNP, and those with an F value <10 were removed for weak strength. Finally, in an effort to identify potential vertical pleiotropic pathways that may arise from specific microbiotic metabolites, multivariable Mendelian randomization (MVMR) analyses were performed using MVMR_IVW to estimate the causal effect of specific gut microbiota on IPF after adjusting for fatty acids.  In addition, the IVW Q statistic[21] and MR-PRESSO global test[27] were utilized to identify horizontal pleiotropic outliers and quantify heterogeneity. The absence of pleiotropic effects was determined if the intercept did not significantly deviate from 0 (p>0.05). Furthermore, a leave-one-out analysis was conducted to identify potentially influential SNPs. The usage and interpretation of our MR study adhere to the STROBE-MR (Strengthening the Reporting of Observational Studies in Epidemiology-Mendelian Randomization) checklist[28] (Supplemental Table S1).  All statistical analyses were undertaken using the “TwoSampleMR” and “MR-PRESSO” packages in R version 4.3.1 (http://www.r-project.org/), and a two-tailed p value of less than 0.05 was considered statistically significant. |
|  | d) | For each exposure, outcome, and other relevant variables, describe methods of assessment and diagnostic criteria for diseases |  | The diagnosis of IPF was conducted based on the guidelines established by the American Thoracic Society and the European Respiratory Society[22, 23] |
|  | e) | Provide details of ethics committee approval and participant informed consent, if relevant |  | Not applied. |
| 5 | **Assumptions** | Explicitly state the three core IV assumptions for the main analysis (relevance, independence and exclusion restriction) as well assumptions for any additional or sensitivity analysis | 5 | In general, three assumptions must be satisfied when using MR analysis. The first assumption is that IVs are associated with the exposure. The second is that IVs are not associated with observed or unobserved confounder factors. The third is that IVs affect outcome only through exposure. |
| 6 | **Statistical methods: main analysis** | Describe statistical methods and statistics used |  |  |
|  | a) | Describe how quantitative variables were handled in the analyses (i.e., scale, units, model) | 9 | Using MR methods and OR, β |
|  | b) | Describe how genetic variants were handled in the analyses and, if applicable, how their weights were selected | 9 | Given the limited number of available SNPs, we selected SNPs significantly related to the gut microbiota with a loose cutoff of p < 1e-5. Then, significant SNPs were clumped within 10,000 kb at the level of linkage disequilibrium (LD) r2 = 0.01 using the European reference panel of the 1000 Genome Project. In reverse MR analyses, independent SNPs were selected by LD (r2 < 0.01 within 250-kb clumping distance, based on the European reference panel of the 1000 Genome Project) at a compromised signifcant level (1e-5) due to the relatively insufficient variables. |
|  | c) | Describe the MR estimator (e.g. two-stage least squares, Wald ratio) and related statistics. Detail the included covariates and, in case of two-sample MR, whether the same covariate set was used for adjustment in the two samples | 9-11 | The two-sample MR study was incorporated to evaluate the causal links between 211 microbial taxa and IPF and lung function. All SNPs were harmonized between the exposure and the outcome by alleles to ensure the alignment of effect. Steiger_filtering test was utilized to remove SNPs associated with reverse causality. IVW method was opted for the as the primary approach to assess the total causal effect of the exposure on the outcome. This involved minimizing the weighted average variance by combining 2 or more IVs, where each IV's weight was calculated as the inverse of the variance of the effect estimate[21]. In addition to the IVW method, we also conducted supplementary analyses, such as Egger regression (MR Egger), weighted median, and weighted mode. MR‒Egger allowed us to explore the average horizontal pleiotropic effect across IVs[22]. The weighted median will provide a consistent estimate if at least 50% of the weight comes from valid instruments and therefore is robust against instrumental outliers[23]. The weighted mode approach assumes that the most frequently occurring association estimate is unaffected by pleiotropy, meaning that it must correspond to the true causal effect[24]. |
|  | d) | Explain how missing data were addressed |  | Not applied |
|  | e) | If applicable, indicate how multiple testing was addressed | 10 | In an effort to identify potential vertical pleiotropic pathways that may arise from specific microbiotic metabolites, multivariable Mendelian randomization (MVMR) analyses were performed using MVMR_IVW to estimate the causal effect of specific gut microbiota on IPF after adjusting for fatty acids. |
| 7 | **Assessment of assumptions** | Describe any methods or prior knowledge used to assess the assumptions or justify their validity | 9 | IVW, MR-Egger, weighted median, and weighted mode |
| 8 | **Sensitivity analyses and additional analyses** | Describe any sensitivity analyses or additional analyses performed (e.g. comparison of effect estimates from different approaches, independent replication, bias analytic techniques, validation of instruments, simulations) | 10 | In addition, the IVW Q statistic[21] and MR-PRESSO global test[27] were utilized to identify horizontal pleiotropic outliers and quantify heterogeneity. The absence of pleiotropic effects was determined if the intercept did not significantly deviate from 0 (p>0.05). Furthermore, a leave-one-out analysis was conducted to identify potentially influential SNPs. The usage and interpretation of our MR study adhere to the STROBE-MR (Strengthening the Reporting of Observational Studies in Epidemiology-Mendelian Randomization) checklist[28] (Supplemental Table S1). |
| 9 | **Software and pre-registration** |  |  |  |
|  | a) | Name statistical software and package(s), including version and settings used | 11 | All statistical analyses were undertaken using the “TwoSampleMR”, “MR-PRESSO”, and “MVMR” packages in R version 4.3.1 (http://www.r-project.org/), and a two-tailed p value of less than 0.05 was considered statistically significant. |
|  | b) | State whether the study protocol and details were pre-registered (as well as when and where) |  | Not yet. |
|  | **RESULTS** |  | 11-21 |  |
| 10 | **Descriptive data** |  |  |  |
|  | a) | Report the numbers of individuals at each stage of included studies and reasons for exclusion. Consider use of a flow diagram | 11 | After screening for SNPs linked with exposure and removing LD, 2,875 SNPs from 211 taxa were employed as IVs. After harmonizing exposure and outcome alleles, all SNPs from various taxa performing MR analysis were shown in Supplementary Table S2. The conclusive findings between gut microbiota and IPF and lung function were summarized in Table 2-6. |
|  | b) | Report summary statistics for phenotypic exposure(s), outcome(s), and other relevant variables (e.g. means, SDs, proportions) | 11 | See in table 2-6 and supplemental table 2. |
|  | c) | If the data sources include meta-analyses of previous studies, provide the assessments of heterogeneity across these studies | 11 | we employed the most recent and extensive GWAS meta-analysis of European ancestry. |
|  | d) | For two-sample MR:  i. Provide justification of the similarity of the genetic variant-exposure associations between the exposure and outcome samples  ii. Provide information on the number of individuals who overlap between the exposure and outcome studies | 9 | To ensure minimal overlap with IPF studies, we specifically selected studies with no or minimal sample overlap. After calculation, we obtained a maximum overlapping rate of <10%, which may not have been sufficient to affect our results[16]. |
| 11 | **Main results** |  |  |  |
|  | a) | Report the associations between genetic variant and exposure, and between genetic variant and outcome, preferably on an interpretable scale | 11-21 | See in Table2-7 |
|  | b) | Report MR estimates of the relationship between exposure and outcome, and the measures of uncertainty from the MR analysis, on an interpretable scale, such as odds ratio or relative risk per SD difference | 11-21 | See in supplemental table 2 |
|  | c) | If relevant, consider translating estimates of relative risk into absolute risk for a meaningful time period |  | Not applied. |
|  | d) | Consider plots to visualize results (e.g. forest plot, scatterplot of associations between genetic variants and outcome versus between genetic variants and exposure) |  | Not applied. |
| 12 | **Assessment of assumptions** |  |  |  |
|  | a) | Report the assessment of the validity of the assumptions | 11-21 | Additionally, we conducted several sensitivity analyses to determine potential heterogeneity and horizontal pleiotropy(Table 2-5). |
|  | b) | Report any additional statistics (e.g., assessments of heterogeneity across genetic variants, such as *I^2^*, Q statistic or E-value) | 11-21 | See in supplemental Table 2-5 |
| 13 | **Sensitivity analyses and additional analyses** |  |  |  |
|  | a) | Report any sensitivity analyses to assess the robustness of the main results to violations of the assumptions | 11-21 | Cochrane’s Q test revealed that there was heterogeneity (Q_pval<0.05). |
|  | b) | Report results from other sensitivity analyses or additional analyses | 11-21 | MR-PRESSO test and MR-Egger intercept tests identified no pleiotropy or significant outliers (P>0.05). |
|  | c) | Report any assessment of direction of causal relationship (e.g., bidirectional MR) | 18-19 | To understand the consequences of IPF and lung function on the abundance of the gut microbiome, a reverse two-sample MR tests were performed. Due to the insufficient number of analyzable SNPs when considering IPF and FEV1/FVC as exposure factors, further MR analysis and subsequent heterogeneity analyses cannot be conducted. Therefore, the reverse MR analysis in this study only focuses on of the casual effects of FEV1 and FVC on the abundance of gut microbiota.  The increase of FEV1 can amplify the abundance of Phylum Actinobacteria (β=3.611, se=0.359, p=0.000), Class Actinobacteria (β=4.554, se=0.623 p=0.000), Order Bifidobacteriales (β=4.812, se=0.558, p=0.000), Family Bifidobacteriaceae (β=4.812, se=0.558, p=0.000), Genus Bifidobacterium (β=4.923, se=0.540, p=0.000), and Genus Ruminiclostridium9 (β=2.445, se=0.426, p=0.000). When investigating the impact of FVC on gut microbiota, a promoting trend for the abundance of the same gut microbiota was observed. |
|  | d) | When relevant, report and compare with estimates from non-MR analyses |  | Details in discussion |
|  | e) | Consider additional plots to visualize results (e.g., leave-one-out analyses) | 11-21 | The leave-one-out sensitivity analysis confirmed that the effect of specific gut microbiota on IPF risk might be driven by some individual SNPs. |
|  | **DISCUSSION** |  | 21-29 |  |
| 14 | **Key results** | Summarize key results with reference to study objectives | 21-22 | In this study, using the summary statistics of gut microbiota from the largest GWAS meta-analysis conducted by the MiBioGen consortium[15] and the latest summary statistics of IPF[17], FEV1, FVC and FEV1/FVC[20] limited to European ancestry, we performed a bidirectional two-sample MR analysis to evaluate the causal association between gut microbiota and IPF and lung function. Six taxa were found causally associated with the risk of IPF. Order Bifidobacteriales, Family Bifidobacteriaceae, Family Prevotellaceae, Genus Holdemania, and Genus RuminococcaceaeUCG009 exerted protective effects on IPF, while Genus Coprococcus2 promote the development of IPF. 23 taxa were causally associated with lung function. Among them, the most prominent beneficial microbiota comprised by Class Deltaproteobacteria, Order Desulfovibrionales, Family Desulfovibrionaceae, Genus FamilyXIIIAD3011group, Genus Fusicatenibacter, and Genus Turicibacter. Meanwhile, Family Lachnospiraceae, Genus Butyricimonas, Genus Lachnospira, Genus Oscillospira, Genus Parasutterella, Genus Senegalimassilia, and Genus Terrisporobacter were associated with the impairment of lung function. In the reverse MR analysis, the abundances of Order Bifidobacteriales, Family Bifidobacteriaceae, and Genus Bifidobacterium increased with the improvement of FEV1 and FVC. |
| 15 | **Limitations** | Discuss limitations of the study, taking into account the validity of the IV assumptions, other sources of potential bias, and imprecision. Discuss both direction and magnitude of any potential bias and any efforts to address them | 29 | There are also limitations to consider. First, the population under study is limited to individuals of European ancestry, therefore, the findings of this study cannot be extrapolated to populations of other ancestral backgrounds. Given the epidemiological variability of IPF prevalence, further research is needed to determine if regional or racial disparities exists. Second, the existence of heterogeneity may lead to inconsistent results and limit the conclusions that can be drawn. We cannot yet demonstrate the underlying mechanism because the goal of our research focuses on correlation analysis. Therefore, further researches are required to explore the specific mechanisms of the lung-gut axis in IPF. Because the feces of IPF patients can be affected by factors other than the disease itself, such as seasonality, age, living habits, diet structure, genetic background, and treatment regimens, it is essential to conduct comprehensive study on the lung-gut axis so that we can provide new directions and strategies for the prevention and treatment of IPF. |
| 16 | **Interpretation** |  |  |  |
|  | a) | Meaning: Give a cautious overall interpretation of results in the context of their limitations and in comparison with other studies | 30 | In summary, the current study suggested the casual effects of the specific gut microbes on the risk of IPF and lung function. In turn, the lung function also exerted a positive role in some gut microbes. Our findings provided novel insights on the potential role of gut microbiota for IPF and indicated a potential mechanism of gut microbiota-mediated prevention of IPF. The MVMR results suggested that fatty acids (monounsaturated fatty acids, total fatty acids, saturated fatty acids, and ratio of omega-6 fatty acids to total fatty acids) probably played a role in the genetic pathway from the gut microbiota to IPF, especially for Bifidobacteriales. |
|  | b) | Mechanism: Discuss underlying biological mechanisms that could drive a potential causal relationship between the investigated exposure and the outcome, and whether the gene-environment equivalence assumption is reasonable. Use causal language carefully, clarifying that IV estimates may provide causal effects only under certain assumptions | 22-28 | Therefore, bacteria that produce lactic acid might also contribute to the progression of IPF. However, a different conclusion drawn by wang and colleagues[41] was that Lactobacillus mucosae can regulate immune responses and intestinal micro-ecological balance by reducing the proportions of inflammatory cells, including granulocytes and monocytes in the blood, and increasing interferon (IFN)-β, interleukin (IL)-1β, IL-10, and tumor necrosis factor (TNF)-α levels. |
|  | c) | Clinical relevance: Discuss whether the results have clinical or public policy relevance, and to what extent they inform effect sizes of possible interventions | 26-28 | There are very close correlations between distinctive gut microbiota and metabolites under pulmonary fibrotic pathological conditions. Short-chain fatty acids (SCFAs), as fecal microbiota transplantation (FMT) metabolic byproducts, are a class of fatty acids produced by the fermentation of dietary fiber by gut bacteria. They typically have a carbon chain length ranging from 2 to 6 carbon atoms. Common SCFAs include acetic acid, propionic acid, and butyric acid. SCFAs have multiple important physiological functions and benefits in the gut, including: (1) Nutrient Source: As products of gut bacteria fermentation of dietary fiber, SCFAs can provide energy and nutrients for intestinal cells. (2) Gut Health: Butyric acid is considered a primary energy source for intestinal mucosal cells. It can promote gut epithelial cell growth and repair, maintain gut barrier function, and reduce gut permeability. (3) Immune Regulation: SCFAs can modulate the functioning of the immune system, promoting the development and function of immune cells, enhancing immune responses, and exhibiting inhibitory effects on the inflammatory process[43]. |
| 17 | **Generalizability** | Discuss the generalizability of the study results (a) to other populations, (b) across other exposure periods/timings, and (c) across other levels of exposure | 29 | The population under study is limited to individuals of European ancestry, therefore, the findings of this study cannot be extrapolated to populations of other ancestral backgrounds. |
|  | **OTHER INFORMATION** |  |  |  |
| 18 | **Funding** | Describe sources of funding and the role of funders in the present study and, if applicable, sources of funding for the databases and original study or studies on which the present study is based | 31 | This study was supported by Shanxi Province science and technology cooperation and exchange special project (Regional cooperation project) (202204041101031), Research Project Supported by Shanxi Scholarship Council of China (2023-190), and Fund Program for the Scientific Activities of Selected Returned Overseas Professionals in Shanxi Province ((2014) 779). |
| 19 | **Data and data sharing** | Provide the data used to perform all analyses or report where and how the data can be accessed, and reference these sources in the article. Provide the statistical code needed to reproduce the results in the article, or report whether the code is publicly accessible and if so, where | 31 | The authors would like to thank the networks for providing the data by MiBioGen consortium (www.mibiogen.org), MRC-IEU OpenGWAS project (<https://gwas.mrcieu.ac.uk/>), the Collaborative Group of genetic studies of IPF (https://github.com/genomicsITER/PFgenetics#study2), and GWAS Catalog (https://www.ebi.ac.uk/gwas/). |
| 20 | **Conflicts of Interest** | All authors should declare all potential conflicts of interest | 32 | The authors declare that the research was conducted in the absence of any commercial or financial relationships that could be construed as a potential conflict of interest. |
